# Design, Modeling, Control, and Application of Everting Vine Robots

**DOI:** 10.3389/frobt.2020.548266

**Published:** 2020-11-10

**Authors:** Laura H. Blumenschein, Margaret M. Coad, David A. Haggerty, Allison M. Okamura, Elliot W. Hawkes

**Affiliations:** ^1^Mechanical Engineering, Purdue University, West Lafayette, IN, United States; ^2^Mechanical Engineering, Stanford University, Stanford, CA, United States; ^3^Mechanical Engineering, University of California, Santa Barbara, Santa Barbara, CA, United States

**Keywords:** tip-extending robot, soft robot, soft actuator, mechanism design, continuum robot, everting robot, vine robot

## Abstract

In nature, tip-localized growth allows navigation in tightly confined environments and creation of structures. Recently, this form of movement has been artificially realized through pressure-driven eversion of flexible, thin-walled tubes. Here we review recent work on robots that “grow” via pressure-driven eversion, referred to as “everting vine robots,” due to a movement pattern that is similar to that of natural vines. We break this work into four categories. First, we examine the design of everting vine robots, highlighting tradeoffs in material selection, actuation methods, and placement of sensors and tools. These tradeoffs have led to application-specific implementations. Second, we describe the state of and need for modeling everting vine robots. Quasi-static models of growth and retraction and kinematic and force-balance models of steering and environment interaction have been developed that use simplifying assumptions and limit the involved degrees of freedom. Third, we report on everting vine robot control and planning techniques that have been developed to move the robot tip to a target, using a variety of modalities to provide reference inputs to the robot. Fourth, we highlight the benefits and challenges of using this paradigm of movement for various applications. Everting vine robot applications to date include deploying and reconfiguring structures, navigating confined spaces, and applying forces on the environment. We conclude by identifying gaps in the state of the art and discussing opportunities for future research to advance everting vine robots and their usefulness in the field.

## 1. Introduction

Growth via tip extension is a form of movement seen in nature across scales and kingdoms, from single-cell pollen tubes (Steer and Steer, 1989) and micro-scale hyphae (Lew, 2011) to creeping vines (Weigel and Jürgens, 2002) and the proboscises of certain worms (Zuckerkandl, 1950; Gibson, 1977). Tip growth has recently been replicated in a variety of robotic systems, referred to as “growing robots” or “vine robots,” using a range of techniques. In addition to tip extension, vine robots are characterized by length change of many thousands of percent and control of their growth direction. We have worked extensively with one method for creating tip extension: pressure-driven “eversion” (i.e., turning inside out) of flexible, thin-walled material. We refer to robots that move in this way as “everting vine robots.”

In this paper, we review much of the existing work on everting vine robots. We discuss the tradeoffs in everting vine robot designs, including materials, actuation, and payloads. We describe the existing quasi-static, kinematic, and force-balance models of growth and steering, and the range of control strategies, from autonomous to teleoperated, that have been implemented. We also describe the important functions and wide range of application of everting vine robots. We conclude by identifying gaps in existing everting vine robot research and highlighting important opportunities for future research. While this paper focuses primarily on our research groups' work on everting vine robots, other groups have contributed to the everting vine robot literature, and their work is referenced throughout the paper where appropriate. Our website, www.vinerobots.org, shares everting vine robot designs and maintains a repository of relevant research.

## 2. Growth and Eversion

Vine robots move via tip extension, which is similar to some forms of biological growth and distinct from locomotion or other animal-like whole body movements. Whereas, movement strategies like locomotion are defined by translation of the body from one location to another (Alexander, 2003), movement by tip extension functions by lengthening the body (Goriely, 2017), reducing or completely eliminating the need to translate relative to the environment.

### 2.1. Bioinspiration

**Figure 1:**
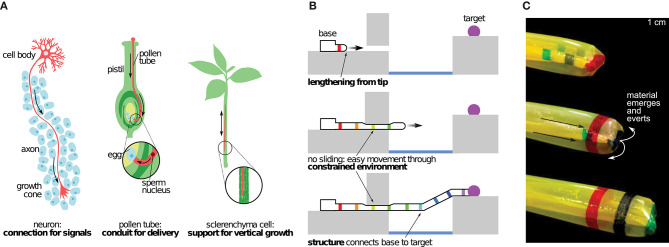
The biological inspiration for and basic properties of tip growth, and our implementation of artificial growth via eversion. **(A)** Examples of biological systems that grow to navigate their environments. **(B)** Schematic representing growth by tip-extension. **(C)** Artificial growth created by pressure-driven eversion of a flexible, thin-walled tube. Modified from Hawkes et al. (2017). Reprinted with permission from AAAS.

The term “growth” refers to a variety of phenomena found in nature, where organisms add mass to their forms. Depending on the exact function, this growth can be an increase in volume (bulk growth), in surface area (accretive growth), or in length (tip growth) (Goriely, 2017). Tip growth (Figure 1A) is often used by systems with non-deterministic body forms to explore their environments and react to changing stimuli. This form of growth is used in nature by a wide variety of plants, animals, and cells to connect locations, deliver payloads, support construction, and more (Sanati Nezhad and Geitmann, 2013). During tip growth, new material is added only in a small region at the tip of a filament (Goriely, 2017). Neurons grow through constrained tissue to create structures that act as signal pathways (Dent and Gertler, 2003). Pollen tubes grow through pistil tissue to build conduits to deliver sperm to the ovary (Palanivelu and Preuss, 2000). Sclerenchyma cells grow within the xylem and phloem to create supporting structures (Sanati Nezhad and Geitmann, 2013). Tip growth is utilized across scales, ranging from the micron scale of fungal hyphae (Lew, 2011), to the millimeter scale of invertebrates that deploy invaginated appendages (Zuckerkandl, 1950), to the centimeter scale of vines and plant roots (Weigel and Jürgens, 2002; Vaughn et al., 2011; Gerbode et al., 2012; Manca, 2018). Through tip growth, these organisms rely minimally on their past states, and instead can pursue evolving nutrient gradients without reconfiguring their bodies.

Such a mechanism for movement is a potentially rich source of bioinspiration in the field of robotics, due to its inherent ability to adapt to complex situations. While traditional robots are effective in controlled settings, and soft end-effectors enhance their ability to interact with a variety of objects, leveraging embodied intelligence for exploration and interaction with dynamic environments remains an open challenge.

### 2.2. Growth in Robots

Replicating elements of biological tip growth, henceforward referred to as “growth,” in robotic systems, i.e., vine robots, has two main benefits (Figure 1B). First, because only the tip moves, there is no relative movement of the body with respect to the environment. This means growth allows for easy movement through constrained environments. Second, as the tip moves, the body forms into a structure in the shape of the tip's path, which can be used for payload delivery, force transfer or self-support, and physical construction. Unlike locomotion, which depends on the reaction forces and mechanical properties of the environment, growth allows vine robots to transfer forces through their bodies, back to their fixed base. Therefore, forces can be generated independent of the contact conditions between the robot tip and the local environment.

Several methods of creating vine robots have been explored thus far. Nested flexible continuum arms have been extended to resemble growth of thin filament structures without concentrating the growth to the tip (Wooten and Walker, 2015). Tip-localized 3D printing has been demonstrated to irreversibly build a robot structure much like in a plant root (Sadeghi et al., 2017). Stored material can be reversibly extended in a variety of ways to replicate the natural behavior of growth, including pulling a chain of rigid links from base to tip (Yan et al., 2019), pulling flexible material from base to tip (Tsukagoshi et al., 2011; Talas et al., 2020), and unreeling flexible material stored at the tip (Dehghani et al., 2017; Satake et al., 2020). Eversion is a particularly elegant method of imitating growth, and it is inspired by mechanisms found in some animals, like the extendable proboscises of certain worms (Zuckerkandl, 1950; Gibson, 1977).

### 2.3. Eversion Growth

Eversion, the opposite of inversion, is the process by which the material internal to a structure turns inside out and becomes part of the outside of the structure. Eversion has been used in toroidal robots to create whole skin locomotion (Hong et al., 2009), imitating cytoplasmic streaming in amoebas, as well as to create a grasping behavior during inversion (Zhu et al., 2016; Li et al., 2020). Everting vine robots achieve growth through pressure-driven eversion of flexible, thin-walled material (Figure 1C). Unlike toroidal robots, which continuously recycle material, an everting vine robot holds one end of its body fixed, while internal pressure effectively pulls the material through the body to the other end. This material everts at the robot tip, resulting in an increase in length. By using pressure-driven eversion of pre-manufactured material, everting vine robots are able to achieve movement by growth to arbitrary lengths, at speeds equivalent to animal locomotion. Additionally, everting vine robots can continue moving even when encountering gaps smaller than their body diameter.

## 3. Design

While the underlying principle of growth through pressure-driven eversion is shared by all everting vine robot designs, the implementation varies. These differences in design, produced by the choice of materials, growth and steering actuation methods, and payload deployment systems, result in different behaviors that must be carefully considered given a desired application.

### 3.1. Materials and Manufacturing

The materials available to manufacture the main body tube of an everting vine robot are confined to those that are inextensible enough to produce eversion as opposed to radial expansion upon pressurization and that are both fluid impermeable and sealable, such that a closed pressure vessel can be developed. Everting vine robot manufacturing techniques are largely material and configuration dependent. While specific designs can necessitate complex and labor intensive manufacturing processes, most everting vine robots are constructed in few steps. In the simplest of cases, an everting vine robot can be constructed by sealing one end of a tube and inverting this sealed end inside the rest of the body (detailed instructions can be found at www.vinerobots.org). This section describes a variety of materials often employed in everting vine robot research and presents the manufacturing methods for each. A summary of these considerations is presented in Table 1.

**Table 1:** Various materials used in everting vine robot designs, with their key behaviors and manufacturing methods.

**Material**	**Key behaviors**	**Manufacturing method**
Thermoplastics (LDPE, TPU)	Fastest prototyping Material uniformity Low burst pressure	Heat sealing/preformed
Thermosets (latex, silicone)	Slow prototyping Variable burst pressure Low hysteresis	Casting/preformed
Thermoplastic-coated fabrics (TPU-coated nylon)	Fast prototyping Moderate burst pressure Good structural characteristics	Heat sealing
Thermoset-coated fabrics (silicone-infused nylon)	Slow prototyping High burst pressure Lowest eversion friction Extensible/inextensible	Adhesives
Uncoated fabrics (ballistic nylon)	Slow prototyping High structural strength High eversion friction	Sewing with internal bladder Ultrasonic welding

*Material choice presents tradeoffs in ease of manufacture, strength, stiffness properties, and actuation pressure*.

#### 3.1.1. Materials Overview

##### 3.1.1.1. Thermoplastics

Thermoplastics are the easiest materials with which to prototype everting vine robots. These off-the-shelf films come manufactured in sheets or tubes, and the two main films used in everting vine robot construction have been low density polyethylene (LDPE) (Hawkes et al., 2017) and thermoplastic polyurethane (TPU). LDPE has an elastic strain limit on the order of 5% (Xu et al., 2016), while the elastic strain limit of TPU is on the order of 50% (Lee et al., 2009). These materials are lightweight, airtight, and inert with respect to most liquids. However, LDPE fatigues easily, often failing after a moderate number of repeated eversions (on the order of 10–50). LDPE is generally purchased in rolls of preshaped tube, and devices are constructed by simply cutting this tube to length and heat sealing the distal end. TPU, however, is often available only as a film, so the film needs to be formed into a tube first, generally through heat sealing. Depending on the application, TPU may also need to be sheathed in a strain-limiting fabric to control radial expansion.

##### 3.1.1.2. Thermosets

Some thermosets, like latex and silicone, can be used, though whether they primarily grow or strain depends on their modulus of elasticity and thickness. These materials are difficult to prototype with, often requiring a strain-limiting layer to evert properly. However, they do have very low hysteresis, and the burst pressure can be controlled by choosing the material stiffness. Thermoset everting vine robots can be manufactured from sheets of thermoset using latex or silicone adhesives or they can be directly cast from liquid silicone into the needed shape.

##### 3.1.1.3. Coated fabrics (thermoplastic and thermoset)

More robust everting vine robots can be built from a variety of fabrics, the most common of which are fabrics coated to be airtight. Everting vine robots constructed from these fabrics can often withstand higher pressures and therefore loads, and they do not fatigue as easily as their plastic counterparts. The woven structure of fabrics also prevents the propagation of holes, thereby reducing the potential for catastrophic failure and allowing for continued operation, assuming the pressure source can provide sufficient airflow to overcome leaks. Thermoplastic-coated fabrics, like TPU-coated ripstop nylon (Coad et al., 2020a), improve the durability of everting vine robots over thermoplastics alone. However, they can suffer from delamination of the thermoplastic layer from the fabric at stress concentrations, resulting in leaks. Other coated fabrics used in everting vine robots include thermoset-coated fabrics, like the silicone-infused ripstop nylon used by Haggerty et al. (2019) and Naclerio and Hawkes (2020). These fabrics do not suffer from delamination but do require different manufacturing techniques than thermoplastics. Silicone-infused ripstop nylon additionally has a low self-friction and, therefore, a much lower required pressure to evert (section 4.1) compared to TPU-coated ripstop nylon, making it desirable for long or very small robots.

Everting vine robots made from coated fabrics are generally manufactured using adhesion methods specific to the coating. For thermoplastic-coated fabrics (e.g., TPU-coated ripstop nylon), the coating is generally on a single side, so the fabric is joined into a tube using an “abutted” joint, i.e., a joint where the single coated side of the material contacts itself. This joint can then be heat-sealed as described above for thermoplastics. For thermoset-coated fabrics (e.g., silicone-infused ripstop nylon), the fabric coating is double-sided, so a tube can be formed using the stronger “lap” joint, i.e., a joint where the opposite sides of the material touch, as described in Naclerio and Hawkes (2020). This joint can be sealed using silicone-based adhesives with light pressure application to ensure a continuous bead of adhesive between the two layers of fabric. The end of the tube can be sealed using a similar method or knotted closed.

##### 3.1.1.4. Uncoated fabrics

Uncoated fabrics, like ballistic nylon, are not airtight, but they have many of the desirable properties of coated fabrics and can be used as a shell for thermoplastic everting vine robots to greatly increase their structural strength. Ballistic nylon was demonstrated in this form-factor for a soft robot without growth in Usevitch et al. (2020). To manufacture an everting vine robot with an uncoated fabric layer, matching tubes of fabric and an airtight layer like thermoplastic (TPU or LDPE) are manufactured. The fabric does not need to be airtight, so it can be sewn together with an abutted seam. An additional seam sewn at the distal end of the robot, passing through the fabric and through the airtight bladder beyond the end seal, can be used to join the two layers. While not necessary, spray adhesive can also be used to form a bond between the two layers along the full length.

#### 3.1.2. Material Extensibility

In addition to the specific class of material, an important design consideration across material type is material extensibility. Soft robotics generally is concerned with using selective strain to produce a specified behavior; soft grippers and crawlers are prime examples of this (Rus and Tolley, 2015; Lee et al., 2017). While early work on everting vine robots exclusively used nearly inextensible materials (Hawkes et al., 2017), later work has investigated the novel behaviors and challenges that come with varying the strain properties of everting vine robot material.

Inextensible materials produce relatively high axial stiffness in everting vine robots, enabling everting vine robots to create self-supporting structures and carry payloads. Everting vine robots made with inextensible materials have been used for reconfigurable antennas (Blumenschein et al., 2018a), haptic wearables (Agharese et al., 2018), and manipulators (Stroppa et al., 2020). As shown in Hammond et al. (2017) and Haggerty et al. (2019), assuming inextensibility can simplify modeling (section 4.1). However, high axial stiffness also means that relatively high forces must be applied to bend or buckle the robot body. This can limit the applicability of these everting vine robots in navigation tasks where environmental contact aids in steering but applied forces must be minimized.

Using body materials with directional extensibility allows everting vine robot stiffness to be varied along different axes. Directional extensibility can be created in thermosets using strain limiting layers, and woven fabrics naturally have a “bias,” i.e., unequal strain along different axes relative to the fabric weave or “grain.” Ripstop nylon in particular has nearly no strain in the direction of the fibers but can strain up to 20% along the 45°-axis (Naclerio and Hawkes, 2020). Everting vine robots made out of silicone-infused ripstop nylon exchange high axial stiffness, when the fabric grain is along the robot body's axis (the “unbiased” orientation), for high torsional stiffness, when the fabric bias is along the robot body's axis (the “biased” orientation). However, the fact that extensible materials reduce everting vine robot stiffness along at least one axis limits the ability of such robots to create self-supporting structures and apply force in certain directions.

### 3.2. Actuating Length Change

**Figure 2:**
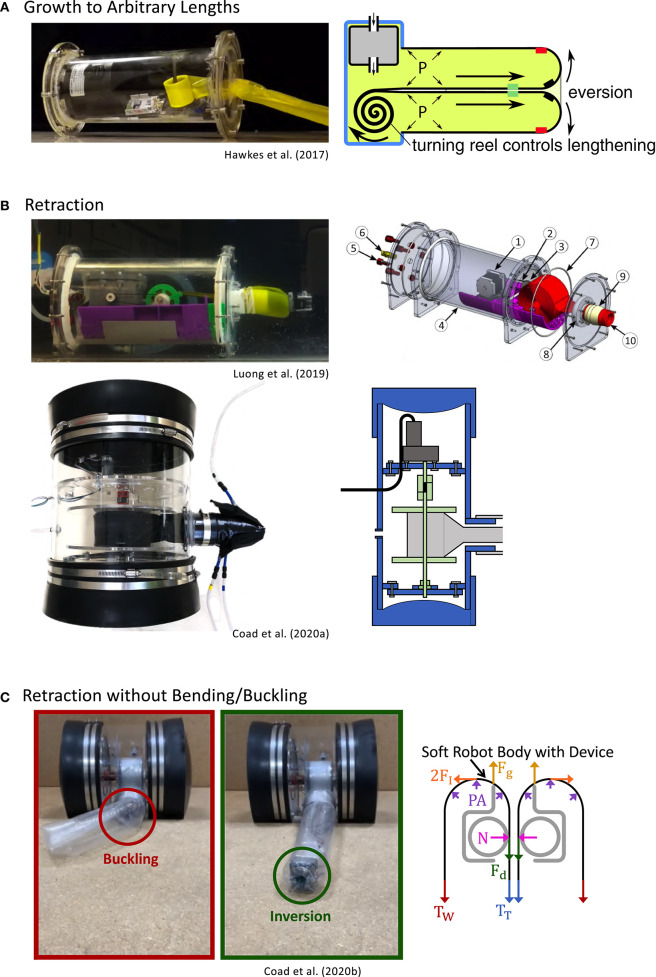
Designs for actuating length change of everting vine robots. **(A)** Storing robot body material on a spool in the base allows growth to arbitrary lengths. **(B)** Reversing the spool direction with a motor allows retraction after growth. **(C)** Adding a retraction device at the robot tip allows retraction without undesired bending or buckling of the robot body. Modified from Hawkes et al. (2017). Reprinted with permission from AAAS. Modified from Luong et al. (2019) © IEEE 2019, Coad et al. (2020a) © IEEE 2020, and Coad et al. (2020b) © IEEE 2020.

Actuation of length change can be considered in two parts: growth, or increasing in length, and retraction, or decreasing in length, both from the tip. Designs for actuating length change are shown in Figure 2.

#### 3.2.1. Growth

Everting vine robot growth is driven by a higher fluid pressure inside the robot body relative to the outside. As growth occurs, the “tail” material travels within the robot body, everts at the robot tip, and becomes part of the robot body wall, i.e., the outer part that moves neither away from nor toward the base.

Depending on the amount of length change that is desired in an everting vine robot, there are two common methods of storing the robot body material before it is everted at the tip. An everting vine robot that doubles in length can be achieved by creating a closed tube of robot body material with a pressure inlet at one end. The tube can be inverted on itself and shortened to half its original length while storing the tail straight inside.

When length change of more than 100% is desired, the robot tail must either be stored in a more compact form or outside the pressurized area of the robot body. Thus far, everting vine robots that store their tail material outside the pressurized area have not been demonstrated in the literature, due to the difficulty of developing an airtight seal through which the tail material can slide during growth, but several everting vine robots have been demonstrated that store the robot tail rolled up on a reel, allowing growth to arbitrary lengths, only limited by the amount of material stored. Hawkes et al. (2017) demonstrated one implementation of this reeled everting vine robot design, where a pressure chamber, the base, was used as a rigid grounding point to attach the robot body wall and a reel of tail material (Figure 2A). Using this design, the robot was demonstrated to grow from a package the size of the base (28 cm) to 72 m long. Provided the base is able to hold pressure needed to grow, the robot length can be scaled arbitrarily.

#### 3.2.2. Retraction

In contrast to growth, in most cases retracting an everting vine robot cannot be accomplished by simply decreasing the relative pressure between the inside and the outside of the robot body. To achieve retraction of the robot body, a force must be exerted on the tail to pull it toward the base while a moderate level of pressure is maintained in the body. Luong et al. (2019) and Coad et al. (2020a) implemented everting vine robot versions where a motor drives the reel in the base, allowing not only control of the material release for growth but also reeling in the material for retraction (Figure 2B).

While this method of retraction works well in a highly constrained environment, everting vine robots retracted in free space tend to bend or buckle into an uncontrollable shape before shortening in length. This uncontrolled behavior, studied in Coad et al. (2020b), is due to the discrepancy between the critical loads for bending or buckling, which are dependent on length, and the force required to invert the material, which is independent of length (see section 4.1 for more discussion of these forces). Thus, above a certain length, an everting vine robot will always bend or buckle rather than retract in a controlled manner. To avoid this problem, Coad et al. (2020b) developed a retraction device (Figure 2C), which sits inside the robot tip and applies the force required to retract the robot body directly to the robot tip, thus making bending or buckling during retraction effectively impossible. When using a retraction device, a motorized reel in the base is still useful to keep slack from building up in the tail and to store the robot body, but the amount of tension on the robot tail can be kept to a minimum (see section 5.1.1).

### 3.3. Actuating Growth Direction

Achieving a desired task with an everting vine robot is often dependent on the ability to dictate the growth direction and the robot shape as it grows and retracts. Here we summarize the different designs investigated to achieve this, while control aspects of everting vine robot steering are discussed in section 5.1.2.

**Figure 3:**
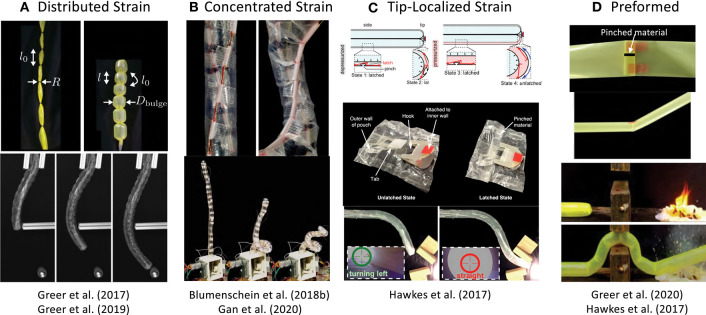
Methods of actuating everting vine robot growth direction and shape, including (top) actuation principles and (bottom) examples of implementation. **(A)** Distributed strain uses pneumatic artificial muscles to create strain along the length where they are attached. **(B)** Concentrated strain uses tendons actuated from the base to change the robot shape. **(C)** Tip-localized strain couples steering and growth to create responsive steering at the tip only. **(D)** Preformed steering shapes the robot for known tasks before deployment. Modified from Greer et al. (2019). The publisher for this copyrighted material is Mary Ann Liebert, Inc. publishers. Modified from Greer et al. (2017) © IEEE 2017, Blumenschein et al. (2018b) © IEEE 2018, and Gan et al. (2020) © IEEE 2020. Modified from Hawkes et al. (2017). Reprinted with permission from AAAS. Modified from Greer et al. (2020).

Steering the everting vine robot body presents a design challenge, since the robot body can grow arbitrarily long. For some applications, the grown length may be less than a meter, while for others, the everting vine robot will be over 10 m in length when in use. Specific design considerations include: the number of actuation inputs needed to sufficiently control the robot shape, the acceptability of uncontrolled robot movements, the scaling of actuator magnitude and speed with the length of the system, and the use of the environment to decrease the required actuation inputs. These design considerations do not have universal answers and often result in application-specific solutions. Generally, actuating growth direction functions by changing the relative length of material on opposite sides of the flexible, thin-walled tube, i.e., shortening or lengthening a side of the tube. Figure 3 shows four different methods of steering everting vine robots, all of which locally shorten or lengthen the robot body material on one side compared to its original length.

#### 3.3.1. Distributed Strain Actuation

One actuation method for steering everting vine robots uses actuators that contract uniformly along their length, so that a single input can uniformly curve the entire robot body. Soft pneumatic actuators are the primary examples of this type of actuation, since the actuator can be long enough to match the full robot length and its compliance allows them to evert with the robot (Figure 3A). All the distributed strain actuators used thus far have been limited in their maximum strain. We quantify this strain using the metric of contraction ratio, defined as the ratio of the difference between shortened and fully extended length to the fully extended length.

Inverse pneumatic artificial muscles (IPAMs), first demonstrated in Hawkes et al. (2016) and used in Zhu et al. (2020) to create fabric muscle sheets, are constructed using a cylindrical rubber bladder enclosed by a strain limiting layer. This layer forces the bladder to expand lengthwise, not radially, when pressurized. IPAMs have been attached to everting vine robots by sewing them into the fabric of the body. Because IPAMs extend at high pressure and contract at low pressure, the robot body needs to be shortened when attaching the IPAMs. Blumenschein et al. (2018a,b) used IPAMs to create helical actuation. Even though these actuators have relatively high maximum contraction ratio (75% was reported in Hawkes et al., 2016), it is difficult to attach IPAMs to an everting vine robot in a way that distributes the strain equally, leading to unpredictable robot shapes.

Unlike IPAMs, both series pneumatic artificial muscles (sPAMs) and series pouch motors (SPMs) shorten when pressurized, making them easy to attach uniformly to an everting vine robot in their unactuated state. These actuators are constructed by creating either radial (sPAMs) or flat (SPMs) constrictions at regular intervals along the length of a tube of airtight, inextensible material. A small space for airflow is allowed through the constriction, yielding a series of small interconnected bubbles or pouches (Niiyama et al., 2015), which shorten lengthwise as they balloon out radially during pressurization. SPMs have a lower maximum contraction ratio than sPAMs [20 vs. 40%, respectively (Greer et al., 2017)], but they are easier to construct and attach to everting vine robots, making them more practical for very long systems. Greer et al. (2017, 2019) demonstrate an everting vine robot steering with 1–2 m long sPAMs, while Coad et al. (2020a) shows steering with 7–10 m long SPMs in a system deployed in the field. The constrictions inherent in these actuator designs can cause drawbacks. They result in high internal fluidic resistance, leading to noticeable time delays in actuation of the more distal segments of a long robot, and they lead to stress concentrations, making the actuators fatigue upon repeated pressurization and depressurization.

Fabric pneumatic artificial muscles (fPAMs) are similar to sPAMs and SPMs but remove the high fluidic resistance. fPAMs are constructed using the bias stretching fabric described in section 3.1.2 formed into a tube with the bias direction oriented along the length of the actuator. When pressurized, fPAMs expand radially and shorten in length, similar to a McKibben actuator (Gaylord, 1958; Geddes et al., 1959). fPAMs were demonstrated in Naclerio and Hawkes (2020) and Selvaggio et al. (2020) to steer everting vine robots. They have a slightly lower maximum contraction ratio (30% was reported in Naclerio and Hawkes, 2020) than sPAMs, but also show very little hysteresis.

#### 3.3.2. Concentrated Strain Actuation

An alternative to distributed strain actuation is concentrated strain actuation. In this category, the actuation comes entirely from the base of the robot instead of distributed along the length, and the actuators are attached only at discrete points on the robot. Generally, concentrated strain actuation has been achieved through tendons routed along the surface of the pressurized tube and pulled by DC motors.

Unlike the pneumatic artificial muscles described in the previous section, actuation using tendons is not inherently strain limited, so tendons can achieve much more dramatic steering. However, the decrease in local stiffness that comes after the onset of local wrinkling of the robot body material (He and Chen, 2014), in addition to the friction that exists in the tendons, means bending due to tendon actuation will concentrate in a single location. This type of actuation was used in Stroppa et al. (2020) to create an approximation of a spherical joint at the base of a growing robot manipulator.

Having all the bending concentrated at a single point can limit the usable actuation scenarios, so other tendon actuation designs include a limit on the local bending. In Blumenschein et al. (2018a,b), Gan et al. (2020), and Wang et al. (2020) this was accomplished through physical hard stops placed along the tendon's routed path on the surface of the tube (Figure 3B). This feature creates a “traveling wave” of bending, with the point most proximal to the base bending first, followed by more distal points as the hard stops connect. While actuating from the base in this way is not generally a better method for steering the tip compared to distributed curvatures from pneumatic artificial muscles, tendon actuation with hard-stops has been used to create complex, well-defined shapes like helices (Blumenschein et al., 2018b) and self-knotting paths (Blumenschein et al., 2020).

#### 3.3.3. Tip-Localized Strain Actuation

The previous two actuation methods show the ability to steer the everting vine robot body independent of growth, so the robot shape can be changed either while at a set length or while growing. However, in these previous methods, more distal portions of the robot body can only be actuated if more proximal sections of the body are as well, limiting the shapes that can be produced. Adding more independently actuated segments along the length of the robot body is possible, but this increases control and design complexity and does not scale well with length. If independent steering control along the full length of the robot as it grows is desired, the robot can instead be actuated by coupling the steering to the growth through tip-localized strain actuation. This has been previously accomplished using preloaded strain that can be released only at the tip, as demonstrated in Hawkes et al. (2017). Mechanical latches hold preloaded strain and can be unlatched when they reach the tip by pressurizing pockets that run along the entire length of the robot (Figure 3C). This couples the steering to the growth, and, as a result, minimizes the actuation signals needed to achieve complex shapes. In 2D, two pressure signals are sufficient to fully shape the robot. A more recent implementation of this actuation method used tensioned strings to pre-load the actuation and servos mounted at the tip to cut the strings as the everting vine robot grew (Cinquemani et al., 2020).

#### 3.3.4. Preformed Actuation

While all the previous actuation strategies created actively controlled robot shapes, active shape change is not needed for some applications. In these cases, the robot body can be pre-formed into the desired final shape before it grows. Two methods have been developed for preforming everting vine robots. In Slade et al. (2017) and Agharese et al. (2018), the robot was shaped by heating the thermoplastic body material (LDPE) while it was stretched over molds of the desired shape. This allowed the material to be heat-set and maintain the shape of the mold once removed, creating smoothly varying shapes. Pinching the body material at discrete points and holding the pinches with pieces of tape creates a similar effect but with discrete turns (Hawkes et al., 2017), which can be seen in Figure 3D.

#### 3.3.5. Passive Environment Steering

**Figure 4:**
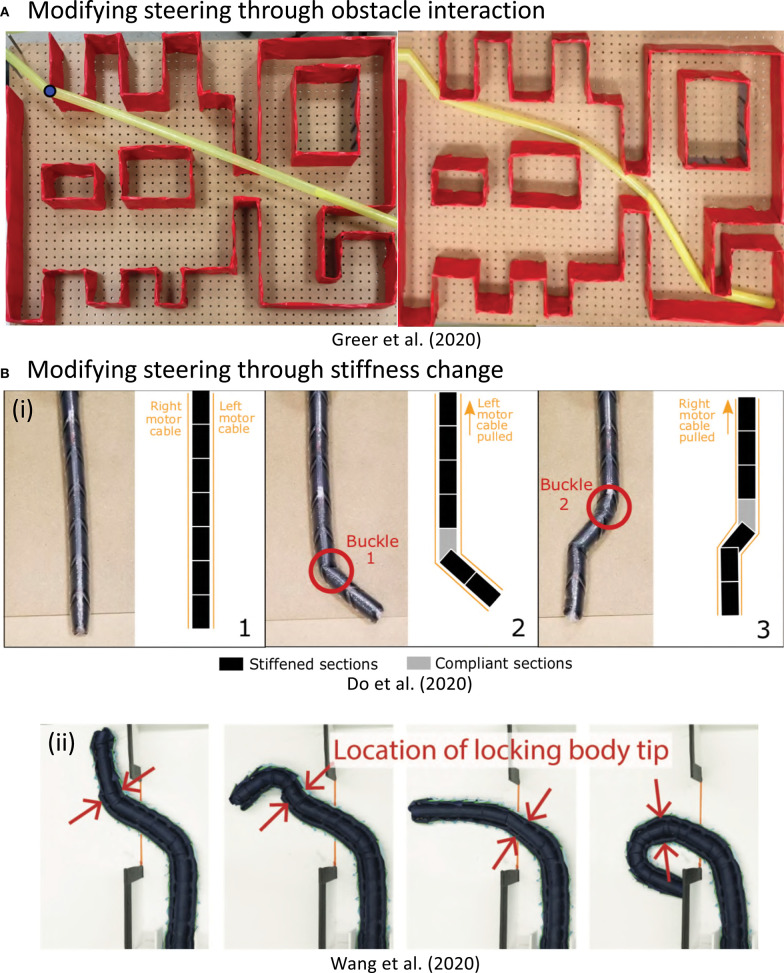
Methods for modifying steering and shape of everting vine robots apart from actuation. **(A)** Steering can be modified by obstacle interaction, where the robot passively conforms to its environment as it grows. **(B)** Steering can also be modified by changing the body stiffness. **(B,i)** Increasing the stiffness of sections through layer jamming allows control of the wrinkling point under tendon actuation. **(B,ii)** Side tubes can be used to shape-lock previous actuation, allowing steering of the tip only and formation of compound curves. Modified from Greer et al. (2020), Do et al. (2020) © IEEE 2020, and Wang et al. (2020) © IEEE 2020.

In addition to creating steering actuation, there are various methods to modify existing actuation, one of which is using the environment to help steer the robot. Everting vine robots can passively adapt to their environment, reaching different final shapes than they would have without environment constraint. Early results of this effect are shown in Hawkes et al. (2017). The compliance and growth behavior of everting vine robots allow them to easily deform around obstacles and follow natural pathways in their environment. Passive steering using the environment was further demonstrated, with heuristic modeling, in Greer et al. (2018). This model was used to design for intentional passive deformations of preformed everting vine robots in Greer et al. (2020) (Figure 4A). The modeling and planning associated with using passive deformation for steering, including using passive deformation with active distributed steering (Selvaggio et al., 2020), will be discussed in sections 4.2 and 5.3.

#### 3.3.6. Stiffness Change

Stiffness change gives a second method of modifying actuation of growth direction and robot shape. As discussed for concentrated strain actuation (section 3.3.2), the local stiffness of inflated tubes rapidly decreases where local wrinkling occurs. Actively increasing the stiffness of the pneumatic tube has recently been investigated to modify this behavior. These designs follow the same considerations as steering actuation: design that minimize the number of control signals and while being scalable with length and remaining flexible enough to allow growth.

Vacuum jamming, i.e., using the frictional forces between particles, lines, or sheets of material to increase the apparent stiffness (Kim et al., 2013), is one method to change stiffness in soft robotic systems. For everting vine robots, Do et al. (2020) showed an implementation of layer jamming that can be used to modify the bending and buckling behavior under concentrated-strain actuation (Figure 4Bi). The passive valves maintain the pressure state of the layer jamming sections and a device traveling inside the everting vine robot body switches the states of those valves (see section 3.4.3 for more discussion of devices inside the robot body).

Stiffness change can also be used to lock previous actuation as the everting vine robot grows, allowing complex robot body shapes to be actuated with only a few actuators. This behavior was achieved in 2D in Wang et al. (2020) using channels on either side of the everting vine robot. Smaller everting vine robots were grown and retracted within these side channels, locking the actuation state of the proximal section of the robot body due to the added friction between the channels and smaller everting vine robots. The distal section of the robot remained steerable via concentrated-strain actuation (Figure 4Bii). This stiffness change design produces behavior similar to that of tip-localized strain actuation, but with the additional ability to reversibly actuate the movement of the distal portion of the robot body.

### 3.4. Mounting Sensors and Tools

**Figure 5:**
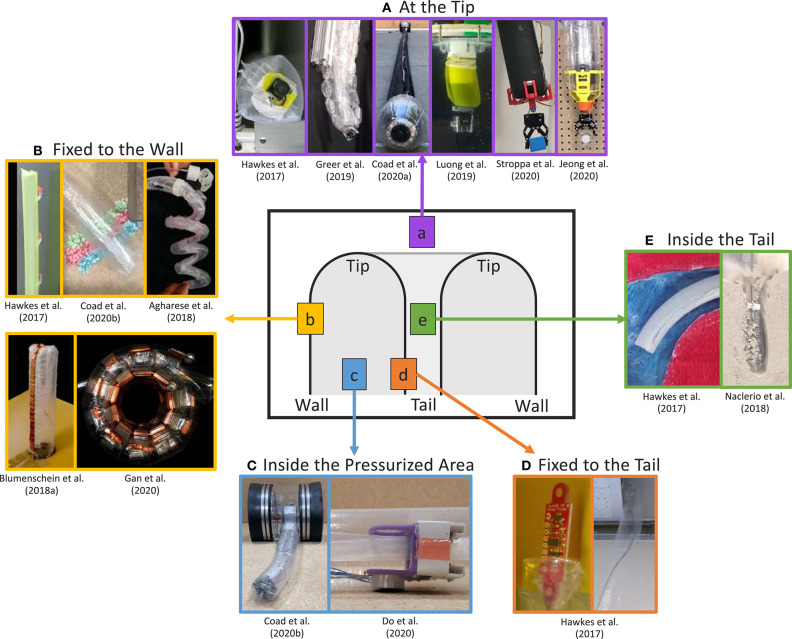
Five locations for mounting sensors and tools around the body of an everting vine robot: **(A)** at the tip, **(B)** fixed to the wall, **(C)** inside the pressurized area, **(D)** fixed to the tail, and **(E)** inside the tail. Modified from Hawkes et al. (2017). Reprinted with permission from AAAS. Modified from Greer et al. (2019). The publisher for this copyrighted material is Mary Ann Liebert, Inc. publishers. Modified from Coad et al. (2020a) © IEEE 2020, Luong et al. (2019) © IEEE 2019, Stroppa et al. (2020) © IEEE 2020, Jeong et al. (2020), Coad et al. (2020b) © IEEE 2020, Agharese et al. (2018) © IEEE 2018, Blumenschein et al. (2018a) © IEEE 2018, Gan et al. (2020) © IEEE 2020, Do et al. (2020) © IEEE 2020, and Naclerio et al. (2018) © IEEE 2018.

Many applications of everting vine robots are made possible by mounting sensors and tools on the robot body and using the robot's movement to transport them through the environment or to reconfigure their shape. Five locations for mounting sensors and tools have been explored thus far and are shown in Figure 5. For some mounting locations, the sensors and tools are fixed to the material of the robot body, and for others, they move in a way that is linked to the robot's movement, but they are not fixed to its material. Key considerations when choosing a mounting location include: how and where sensors and tools need to interact with the environment and how placement will encumber the movement of the everting vine robot.

#### 3.4.1. At the Tip

Because the tip of an everting vine robot is often the first point to enter a new space, this is an important area to mount sensors and tools that interact with the environment (Figure 5A). Sensors mounted at the robot tip, such as a camera (Hawkes et al., 2017; Greer et al., 2019; Luong et al., 2019; Coad et al., 2020a), can be used to sense properties of the environment and to provide feedback of the robot state during navigation and exploration. Meanwhile, tip-mounted tools, such as a gripper (Jeong et al., 2020; Stroppa et al., 2020), enable environment interactions, such as picking up objects and pulling on the environment.

Mounting to the robot tip is challenging, since the specific section of robot body material at the tip continually changes during eversion and inversion. Thus, a tip mount must move relative to the robot body material, not merely be adhered to the material. Jeong et al. (2020) analyzed the various tip mount designs that have been developed and defined design principles for successful tip mounts. The methods by which sensors and tools have been attached to the tip include: cables inside the tail (Mishima et al., 2006; Hawkes et al., 2017; Greer et al., 2019), friction with the wall (Coad et al., 2020a), magnets (Luong et al., 2019; Stroppa et al., 2020), and rolling interlocks (Jeong et al., 2020). Many of these tip mount designs use parts both outside the robot body and inside the pressurized area at the robot tip to stay attached. Wire management is also a challenge because wires must move relative to the robot's body. Luong et al. (2019) showed a wireless tip mount, but previous solutions to manage wired connections have consisted of wires inside the robot tail (Hawkes et al., 2017; Greer et al., 2019) and external wires with a self-sealing zipper pocket to avoid snagging on the environment (Coad et al., 2020a). Mounting at the tip involves a tradeoff between reliable attachment and encumbrance of the everting vine robot's natural ability move through confined spaces. The sensors, tools, and mounting methods can also add large or heavy elements at the robot tip, limiting the everting vine robot's ability to support its own weight, pass through small apertures, and move relative to the environment without friction.

#### 3.4.2. Fixed to the Wall

Another method to directly place sensors and tools in contact with the environment is fixing them to the robot body wall (Figure 5B). This location is well-suited for mounting items that are deployed during growth or that need to interact with the environment along the entire length of the robot body, although anything mounted must be flexible enough or small enough to be everted and inverted along with the robot body material. While this location can be useful for some sensing applications, many of the demonstrated designs have mounted non-traditional robot payloads to the robot body wall. Adhesive patches attached to the outside of the body can be used to grip the environment, in one case to provide additional support when climbing vertically (Hawkes et al., 2017) and in another to takes samples of the environment (Coad et al., 2020b). Items attached to the body can also be deployed and shaped by the robot. Agharese et al. (2018) shows deployment of soft haptic actuators, and Blumenschein et al. (2018a) and Gan et al. (2020) show deploying and shaping segmented antenna pieces in order to form functional devices.

#### 3.4.3. Inside the Pressurized Area

Items that do not need to interact physically with the environment can be mounted inside the pressurized area of the robot body (Figure 5C). The structure of the robot body acts as a pathway which can be traveled independent of the growth of the robot and without contacting the environment. The physical separation from the environment means mounting inside the pressurized area is best suited for sensors and tools used to interact with the robot body itself, or those that can interact with the environment in a non-contact fashion. This mounting location was used in Coad et al. (2020b) to attach the retraction device (section 3.2.2), which applies force to the robot tail to retract the robot body after growth. Similarly, Do et al. (2020) demonstrated a motorized carriage device moving internal to the robot to carry an electromagnet. Wired transmission of power from the base helps reduce device weight. As with wires passed to tip mounts, these wires must span a changing length as the device moves along the robot. so the wires should be managed to keep them taut while reeling them in or out as needed. Mounting inside the pressurized area does not require an active carriage device, as friction with the tail can passively keep devices at the tip during growth. Watson and Morimoto (2020) used this method to keep a ring magnet at the tip of a millimeter-scale everting vine robot for tip-localization.

#### 3.4.4. Fixed to the Tail

Due to eversion, the robot tail moves at twice the speed that the robot tip moves relative to the base. Mounting sensors and tools to the robot's tail is therefore a useful way to transport items between the robot base and the tip, using the growth and retraction of the robot itself (Figure 5D). Items fixed to the inside of the tail can contact the environment once that portion of the tail reaches the robot tip; rather than becoming part of the wall, the items may be deployed into the environment or reach the tip at the fully grown robot length. Hawkes et al. (2017) used this mounting location to demonstrate delivery of items from the robot base to the robot tip during growth through difficult environments. A sensor packaged safely inside the tail was protected from environmental hazards until the very end of growth when it was deployed out into the environment, and a wire was tied to the robot tail and pulled through the inside of the robot body, easily routing the wire through a confined space. The main disadvantage of this mounting location is that the robot length when the payloads will reach the tip is fixed at the time of manufacture. Either the desired final robot length must be known before launching the robot or it must be determined through trial and error.

#### 3.4.5. Inside the Tail

To overcome the disadvantages of fixing payloads to the tail, sensors and tools can be mounted inside, but not fixed to, the robot tail (Figure 5E). Using this mounting location, items can be passed from the base to the tip such that some part of them stays continually at the tip during growth and retraction. As mentioned in the previous subsection, the robot tail and the robot tip move at different speeds relative to the base, so the payload must slide within the robot tail to remain at a desired location. If the everting vine robot material is not stored on a reel, this can be achieved by leaving the end of the tail partially unsealed so that items can pass from outside the base through the tail. However, the internal pressure used to grow the robot will cause the tail to naturally squeeze anything inside it, so some way to balance the pressure, like sending a steady stream of air through the tail, is needed to allow sliding of items inside the tail. Hawkes et al. (2017) used this mounting location to pass a tool through the robot body from base to tip in a demonstration of a medical procedure, while, Naclerio et al. (2018) passed a tube through the tail to the robot tip to send compressed air to fluidize a granular environment and allow the robot to grow through it with ease. While mounting inside the tail is good for passing items through the robot body to the outside of the robot tip, also storing the robot body material on a reel in the base is impossible, because of the need for relative movement between the tail material and the items inside the tail. This provides incentive to find other methods of storing the robot body material compactly when not in use. Additionally, maintaining the appropriate relative speed of movement between the tail material and the items inside such that part of the items remains at the tip is challenging. The items inside the tail need to be pulled toward the base during growth and pushed away from the base during retraction (not yet demonstrated in the literature).

## 4. Modeling

**Figure 6:**
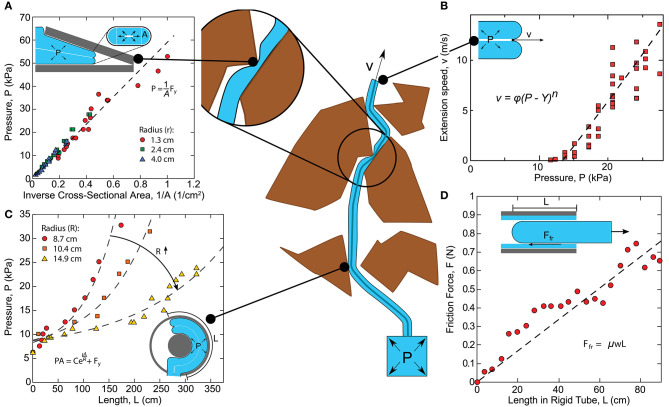
Quasi-static modeling of everting vine robot growth. Model relates the driving force (internal pressure times tip cross-sectional area) to the losses due to the robot state, including **(A)** static yield force (i.e., driving force required to begin growth), **(B)** viscoplastic loss due to everting material, **(C)** exponential friction for moving tail material around curves in path, and **(D)** linear friction as a function of length/weight of tail material being transported. Modified from Blumenschein et al. (2017). Reprinted with permission from Springer Nature.

**Figure 7:**
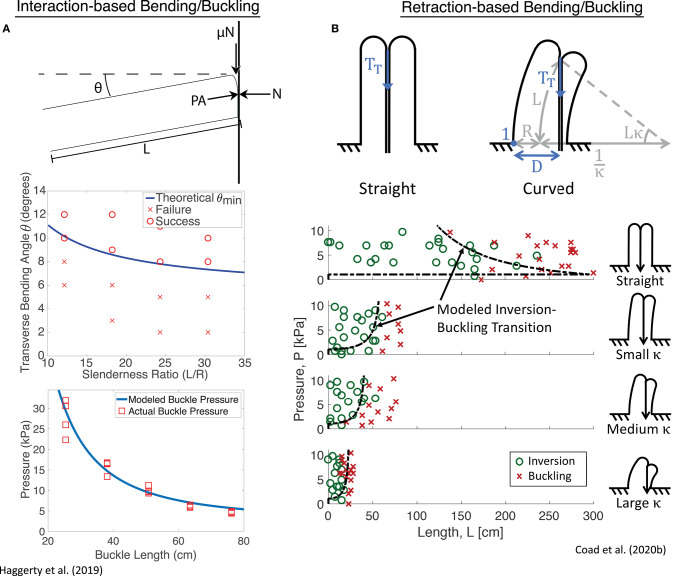
Quasi-static modeling of everting vine robot bending and buckling during growth into obstacles, as well as retraction. **(A)** Modeling of bending and buckling based on environment interaction allows prediction of the pressure required to passively deform through an environment during growth. **(B)** Modeling of bending and buckling based on retraction forces allows prediction of when the robot will invert successfully. Modified from Haggerty et al. (2019) © IEEE 2019 and Coad et al. (2020b) © IEEE 2020.

**Figure 8:**
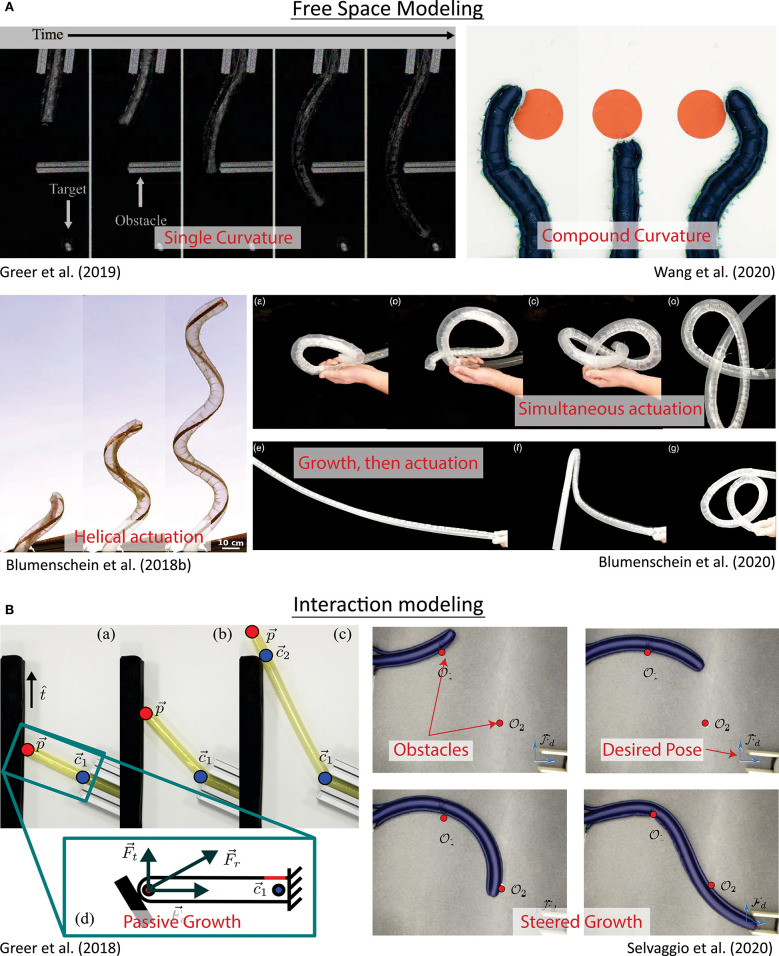
Kinematic and force-balance modeling of everting vine robot shape/steering. **(A)** Modeling of robot shape in free space has included kinematic models based on constant curvature and piecewise constant curvature sections, some of which also consider forces, as well as kinematic models based on helical and piecewise helical actuator routings. **(B)** Modeling of robot shape during environment interaction has developed heuristics for both passively and actively steered growth based on kinematics and force-balance models. Modified from Greer et al. (2019). The publisher for this copyrighted material is Mary Ann Liebert, Inc. publishers. Modified from Wang et al. (2020) © IEEE 2020, Blumenschein et al. (2018b) © IEEE 2018, Greer et al. (2018), Blumenschein et al. (2020) © IEEE 2018, and Selvaggio et al. (2020) © IEEE 2020.

As with many soft robots, everting vine robots present specific challenges for modeling, and even more so because growth is such a unique form of movement. As a result, models for growth and steering of everting vine robots draw inspiration from a variety of sources, including models of other soft robotic systems and models of naturally occurring growth and steering. Even though the method of growth through eversion is unlike many natural systems, the mathematics of biological growth as seen in the literature (Goriely, 2017) has a close link to the models of growth that describe everting vine robots, and the principles that describe how a plant shapes itself, for example, how a cucumber tendril forms a helix (Gerbode et al., 2012), closely relate to the understanding of how differential shortening allows everting vine robots to form similar shapes (Blumenschein et al., 2018b). Section 4.1 outlines the quasi-static analyses conducted to generate models of growth (Figure 6), as well as bending and buckling due to growth into obstacles and due to retraction (Figure 7). Section 4.2 describes the kinematic and force-balance modeling employed to predict robot shape due to both active and passive steering (Figure 8).

### 4.1. Modeling of Growth

An important portion of everting vine robot modeling has focused on understanding everting vine robot growth and retraction, including the forces at play due to interaction with the environment. Thus far, these models have all been limited to quasi-static analyses, i.e., those that neglect dynamics. Many of the analyzed movements were slow enough that dynamics could be discounted, but faster growth movements have also shown negligible inertial effects.

Blumenschein et al. (2017) showed a quasi-static model for growth via pressure-driven tip eversion based on an equilibrium force balance (Figure 6). The model equates the driving force, i.e., the internal pressure multiplied by the tip area, to internal losses. The losses break down into two categories: losses associated with transporting material from the base to the tip, and losses associated with everting new material at the tip. Material transport is dominated by the frictional interaction of the everting vine robot material with itself, due to the weight of the tail material (Figure 6D), and the tension of the inner material being pulled around curves (i.e., the capstan equation, see Lubarda, 2014) (Figure 6C). At the tip, Hawkes et al. (2017) show experimentally that eversion losses closely match the viscoplastic behavior of other pressure-driven growing systems (Figures 6A,B), like the expansion of plant cells (Green et al., 1971) or deployment of invertebrate proboscises (Zuckerkandl, 1950), with a yield force (i.e., a minimum driving force to begin growth) and a viscous damping as a function of growth speed, with negligible inertial effects. This model allows the user to predict whether growth will occur, and at what speed, given the pressure and robot geometry.

Naclerio et al. (2018) and Haggerty et al. (2019) expand on this model by adding the effects of external forces from the environment. In Naclerio et al. (2018), the model was specifically adjusted to account for the resistive forces of the sand on growth during burrowing. Haggerty et al. (2019) focused more broadly on the environmental interaction forces that passively steer an everting vine robot while navigating a cluttered environment through self-buckling or self-bending (section 3.3.5). Simple geometric and pressure dependent models predict bending and buckling for everting vine robots (Figure 7A), largely informed by existing bending and buckling models for inflated beams (Comer and Levy, 1963; Fichter, 1966; Le-van and Wielgosz, 2005). Godaba et al. (2019) further considered the buckling and bending loads to determine payload capabilities, and Putzu et al. (2018) looked into the relationship between force applied to the robot tip in compression and the robot's growth speed.

These bending and buckling behaviors can also occur due to forces applied during retraction (section 3.2.2). Coad et al. (2020b) described the critical points for inversion-based buckling as a function of curvature, length, and internal pressure (Figure 7B). The same length-independent yield force that must be overcome to begin eversion is also required to begin inversion, while the forces required to bend and buckle the robot body decrease with increasing length. This means that regardless of robot curvature and internal pressure, above a certain length, the robot body will always bend or buckle instead of inverting.

### 4.2. Modeling of Steering

Kinematic and force-balance models have been employed to calculate the robot shape both due to actuators and due to obstacle interaction. These models are highlighted in Figure 8.

Early models for everting vine robot steering were inspired by constant curvature models used for flexible-backbone continuum robots (Webster and Jones, 2010). In Greer et al. (2017), constant curvature kinematics are used to define the 3D shape of a flexible, thin-walled inflated backbone, without eversion, steered by distributed strain actuators (Figure 8A). This model incorporates a force balance, taking into account the backbone and actuator stiffnesses due to pressure. Greer et al. (2019) then incorporates the effects of the changing body length when growing. While these effects are mainly accounted for using control strategies (section 5.1.2), it is noted that the change in body length also causes a reduction in the frequency response of the actuators as they increase in length, due to the fluidic resistance of sPAMs (section 3.3.1). Greer et al. (2019) also showed that the mapping between internal actuator pressures and instantaneous tip displacements is fairly consistent throughout the robot's workspace. This allowed Coad et al. (2020a) to develop a simplified kinematic model assuming a linear relationship between change in actuator pressure and instantaneous tip displacement. This model commands instantaneous tip displacements, instead of absolute tip positions.

Adding shape-locking (section 3.3.6) to a robot with constant curvature actuation allows for the creation of complex compound curvatures, but this requires a modification of the constant curvature models as a result. Wang et al. (2020) developed a steering model to determine the tip position of a shape-locking everting vine robot (Figure 8B). This method of shape-locking causes the more proximal sections to be held in place while the most distal section, past the end of the locking bodies, can actuate into a constant curvature shape. The full robot shape is a compound curve made of constant curvature segments. As the locking bodies grow or retract along the robot, new static segments are added or removed from the curve, and the tip position can be reconstructed by taking the kinematics of each curved segment in order.

These constant curvature models only apply to actuators mounted parallel to the backbone, i.e., parallel to the growing direction of the everting vine robot. Blumenschein et al. (2018b) expanded these steering models to actuators attached to the everting vine robot body in a helix (Figure 8A). The developed closed-form kinematics for helical actuators relate the 3D actuator shape to the 3D deformed robot shape based only on geometry. To model the kinematics of general actuator shapes on everting vine robots, Blumenschein et al. (2020) took this helical kinematics model and approximated general paths as piecewise helical. This approximation accurately predicts the actuated shapes resulting from generally shaped actuators. The kinematic modeling was also used to design the actuation to achieve a desired path, like a self-knotting everting vine robot (Figure 8A).

Steering can also result from obstacle interactions. A model presented in Greer et al. (2018) developed a simple kinematic heuristic for a straight (unactuated) everting vine robot as it grows into an obstacle in 2D: the tip will slide along the obstacle in a direction determined by the initial contact angle, and the robot will bend at the previous contact with the environment (Figure 8B). Given an environment including some set of obstacles, this model predicts the robot's path based entirely on the obstacle locations and initial robot state, keeping track of obstacle contact points on the everting vine robot. In Greer et al. (2020), a slight modification of the obstacle interaction model was used to account for preformed turns as well, and this model was used to plan 2D paths through environments with known obstacles (section 5.3).

Active steering and obstacle interaction models can be combined to model controlled everting vine robots moving through obstacle-filled environments. Selvaggio et al. (2020) shows a piecewise formulation to calculate the robot shape during environment contact in 2D. The free length of the robot body (i.e., the section not constrained by the environment) takes on a constant curvature shape determined by the pressures in the actuators, while the constrained length of the robot body is shaped based on the obstacle contact locations (Figure 8B). A point-loaded cantilever inflated beam model determines the deflection and moment of the constrained section of the body. This model can similarly be used for planning (section 5.3).

## 5. Control and Planning

The unique properties and mechanisms of everting vine robot movement provide new opportunities and challenges for robot control and planning, both teleoperated and autonomous. Considerations include what behaviors can be planned and how to bring a human operator into the control loop. The main everting vine robot control and planning topics studied thus far have been (1) robot-level control of growth, retraction, and steering, (2) interface design to allow human operators to teleoperate everting vine robots, and (3) planning methods that consider obstacle interaction models of everting vine robots.

### 5.1. Robot-Level Control

**Figure 9:**
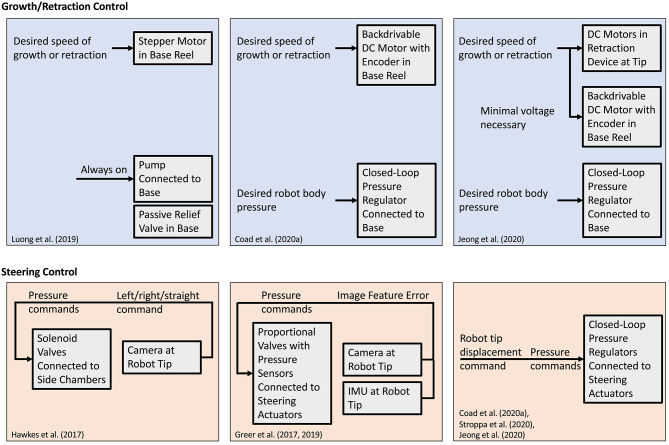
Control schemes for the growth/retraction and the steering degrees of freedom of robot tip movement. **(Top)** Control of growth and retraction speed is achieved by balancing internal pressure with motor inputs to maintain the proper level of tension in the tail. **(Bottom)** Steering control is achieved by mapping desired tip displacements to pressures in steering actuators.

Robot-level control strategies are concerned with controlling the fundamental movements of the everting vine robot. Since growth and steering are generally actuated independently, the control strategies are handled separately as well. Even when steering is coupled to growth, the control of steering is separate and reactive to growth. Control schemes that have been demonstrated in the literature are diagrammed in Figure 9.

#### 5.1.1. Growth and Retraction Control

Due to the variability of length scales of everting vine robots, growth and retraction have been speed controlled. Accurate control of the robot's length relies on being able to apply forces that both lengthen and shorten the robot. Since internal pressure can only drive growth, an antagonistic actuator, like a motor attached to the tail, is needed to have full control. Using the antagonistic combination of pressure to drive growth and motor to resist growth, speed control has been achieved for limited length change (Greer et al., 2019) and arbitrary length change (Luong et al., 2019; Coad et al., 2020a) robots.

The exact implementation of growth control differs between these systems. Luong et al. (2019) used a continuously-running pump with a relief valve to maintain a constant pressure (20 kPa), while growth and retraction speed were controlled via commands sent to a stepper motor. Care was needed to ensure that the stepper motor did not introduce slack if obstacles or steering slowed the robot. Coad et al. (2020a) used a backdrivable DC motor with an encoder and a closed-loop pressure regulator to make growth speed control robust to these disturbances without sensing the true growth speed. By setting the motor to only resist growth and allowing the pressure to backdrive the motor up to the desired speed, the speed could be controlled without allowing slack in the tail.

While retraction can be accomplished with the architecture described above, controlled retraction has been implemented with the addition of a retraction device (Coad et al., 2020b) as discussed in section 3.2. With this device, the motor inputs of the base motor and the retraction device motor(s) must be synchronized and their combination must balance the internal pressure. Jeong et al. (2020) presented an implementation of growth and retraction control using a retraction device without an encoder. The retraction device motors determined the speed of growth or retraction, while the base motor applied the forces necessary to maintain material tension and reel material slack as it developed.

#### 5.1.2. Steering Control

Unlike growth and retraction control, steering control methods are dependent on the actuation method used. This section only discusses steering control when in free space; the steering behavior of everting vine robots under environmental contact is treated as a planning problem instead.

Control for tip-localized strain actuation (section 3.3.3) was demonstrated in Hawkes et al. (2017). Since steering could only occur at discrete points when the robot grew, bang-bang control was used, where the next command–left, right, or straight–was queued according to the target location relative to the tip. This method could stably control the everting vine robot heading as long as the growth speed was sufficiently slow, since actuation inputs occurred at discrete intervals and resulted in irreversible shape change of the body.

Control of reversible steering was first demonstrated in Greer et al. (2017, 2019) with an everting vine robot using distributed strain actuation. Since steering is completely decoupled from growth and retraction, instantaneous movement of the robot tip in any direction is possible. For autonomous control, tip motion from steering was commanded with a visual servo control law to keep tracked features centered in the field of view. Even though an image-space Jacobian could be derived based on constant curvature models (section 4.2), the control instead used model-free approaches and calibrated an image-space Jacobian approximation during startup. The Jacobian translated actuator pressures to image-space displacements. The camera could spin relative to the robot, so an IMU attached to the camera was used to estimate the relative rotation of the tip camera and update the Jacobian.

Coad et al. (2020a) also demonstrated steering control for distributed strain actuators, using a simplified kinematic model of the robot instead of a model-free image-space Jacobian, and for the purposes of teleoperation. This method controlled the robot body at relatively long lengths (7.5–10 m) for the first time, demonstrating that constant curvature assumptions break down at long length. Only the most distal meter long section of the robot body achieves a consistent curvature, so past that length, the kinematics can be considered approximately independent of length. Since human-in-the-loop teleoperation was used to provide reference inputs instead of feedback from a tip camera, the steering control was open-loop and based on the inverse kinematics. This steering control method was also modified to be used with concentrated strain actuation in Stroppa et al. (2020) and was demonstrated with retraction in Jeong et al. (2020), and model-based control using beam bending models was shown in Ataka et al. (2020).

### 5.2. Input Modalities

**Figure 10:**
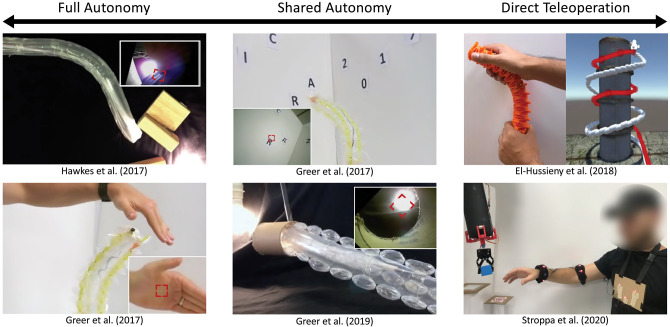
Input modalities for everting vine robot control on the spectrum from full autonomy to direct teleoperation. **(Left)** Full autonomy has been demonstrated for simple tasks, such as following a continuously visible stimulus in the robot's field of view. **(Middle)** Shared autonomy has used a point-and-click interface for the human operator to direct the robot toward a set of waypoints in its camera view. **(Right)** Direct teleoperation has used both off-the-shelf and custom-designed interfaces that are held or worn and used to complete navigation and pick-and-place tasks. Modified from Hawkes et al. (2017). Reprinted with permission from AAAS. Modified from Greer et al. (2019). The publisher for this copyrighted material is Mary Ann Liebert, Inc. publishers. Modified from Greer et al. (2017) © IEEE 2017, El-Hussieny et al. (2018) © IEEE 2018, and Stroppa et al. (2020) © IEEE 2020.

Input modalities refer to the methods used to provide reference commands to the robot (Figure 10). Everting vine robots can be fully or semi-autonomous, relying only on high-level commands from operators and feedback from sensing within their control loop, or they can be directly teleoperated, taking low-level commands from a human operator.

#### 5.2.1. Full and Shared Autonomy

Full and shared autonomy was demonstrated in Greer et al. (2017, 2019) and Hawkes et al. (2017), using a camera and video processing to track image features that are selected by the operator (Figure 10). Full autonomy is possible in cases where the tracked image feature is constant and always in view, allowing the everting vine robot to navigate toward a light in Hawkes et al. (2017) or to follow a person's hand in Greer et al. (2017). When different features need to be tracked over time, either due to changing goals or because the end goal is not in sight, humans can provide updates to the target object in a shared autonomy setup. Shared autonomy was shown in Greer et al. (2017) to switch targets in a sequence, and in Greer et al. (2019) to navigate toward a target hidden behind an obstacle in the workspace.

#### 5.2.2. Direct Teleoperation

A variety of devices have been used to provide inputs for direct teleoperation, including off-the-shelf input devices and custom-designed interfaces. Since growth is a degree of control not found in many robots, a key early consideration for interface design was the intuitiveness of the control. El-Hussieny et al. (2018) conducted a user study of teleoperation using a simulated everting vine robot with first-person view as though from a camera at the robot tip (Figure 10). Three off-the-shelf input devices (keyboard, joystick, and Phantom Omni) were compared to a novel flexible joystick. Overall, the novel flexible joystick outperformed the other input devices on all measured metrics and was found to have the lowest self-rated mental workload. A similar flexible joystick was used in Coad et al. (2020a) for teleoperation of an everting vine robot within a previously unexplored rocky tunnel in an archaeological site. Joystick displacements were mapped to robot tip displacements and the growth speed of the robot was input using a sliding potentiometer embedded in the joystick. The human operator received feedback of the robot tip position by viewing images from a camera at the robot tip. A different interface for direct teleoperation of everting vine robots was demonstrated in Stroppa et al. (2020) for a pick-and-place task (Figure 10). This interface used a motion capture system with markers placed on the human operator's chest and arm, tracking the operator's gestures to control the growth, retraction, and steering of the robot, while the human operator viewed the entire robot body and its environment via direct line of sight. In a user study, participants teleoperated the everting vine robot to successfully transfer a cube from one platform to another in 95% of trials.

### 5.3. Planning

**Figure 11:**
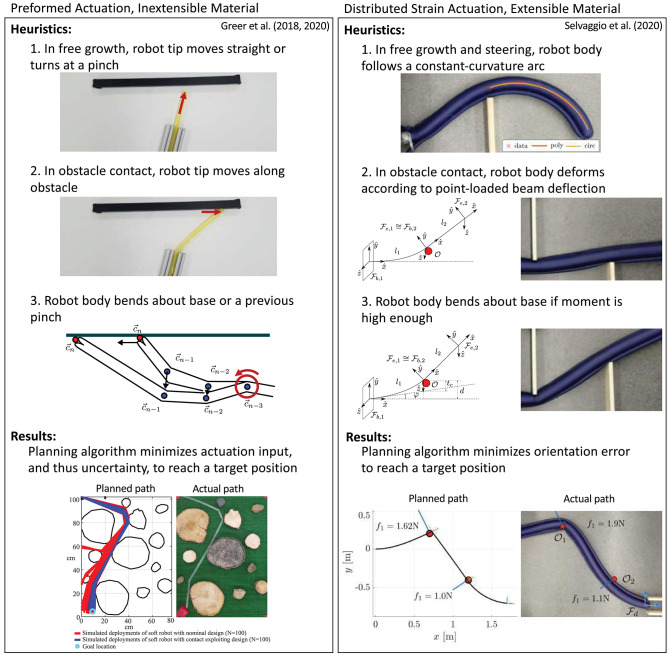
Planning methods for **(left)** a preformed everting vine robot made of inextensible plastic and **(right)** an everting vine robot steered with distributed strain actuators and made of extensible fabric. The planning methods leverage heuristics about robot shape and environment interaction to minimize actuation input or orientation error while reaching a target position. Modified from Greer et al. (2020) and Selvaggio et al. (2020) © IEEE 2020.

Everting vine robots interact with their environment in ways desirable for navigation, creating opportunities for planning methods that are unique to these types of robots. Thus far, the literature has focused on defining and using heuristics for everting vine robot interaction with a known, rigid environment. These planning methods demonstrate that designs that use environmental contact have a higher probability of reaching a target in the face of actuation uncertainty, and that the dexterous range of everting vine robots can be increased by contacting the environment. The planning methods that have been demonstrated in the literature for everting vine robots are shown in Figure 11.

Greer et al. (2020) used the obstacle interaction heuristics for an everting vine robot with preformed steering to develop a planning method for choosing the initial robot shape, i.e., the pinch locations and pinch angles (section 3.3.4). The planning method maximized the probability of reaching a desired target given noise in the design parameters. This planning method uses the certainty of the robot tip position when contacting obstacles to counteract the uncertainty in manufacturing the preformed everting vine robot, as well as offloading some of the manipulation of the robot shape to the environment, reducing the required actuation. To find a plan, a sequence of waypoints overlaid on the known map and linking the start and end while requiring the minimal amount of preformed actuation were identified. Then, from the possible designs, the one that maximizes the probability of reaching each waypoint was selected.

Selvaggio et al. (2020) presents a similar planning method with the addition of active steering. A slightly different model (detailed in section 4.2) is used to describe the obstacle interaction of these robots. This model can calculate the reachable workspace of the robot tip as a function of a sequence of obstacle interactions; the more obstacles that can be used to manipulate the robot's path, the greater the possible range of approach angles of a target location. For a desired approach angle, the planning problem iterates through all possible permutations of obstacle contact states to find the sequence of obstacle contacts that minimize the orientation error at the target.

## 6. Applications

**Figure 12:**
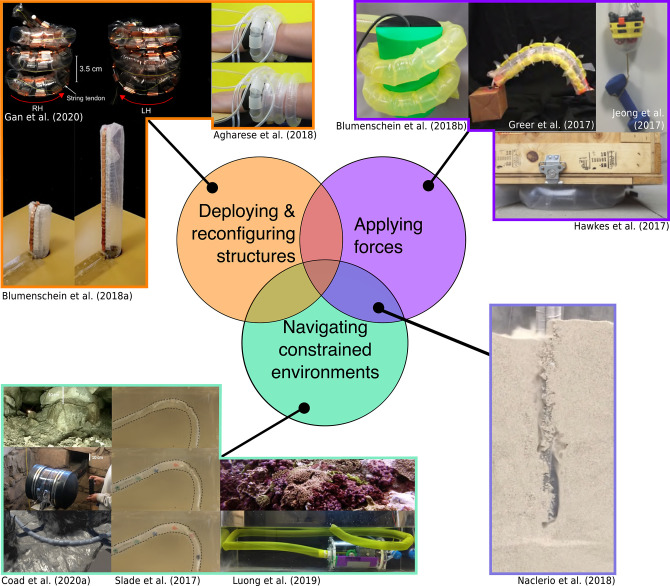
Everting vine robot applications organized by the function of the robot in the application, including **(top left)** deploying and reconfiguring structures, **(bottom left)** navigating constrained environments without damaging the environment or the robot, and **(top right)** applying forces to the environment through squeezing, pushing, pulling, or expanding. **(Bottom right)** Some applications, such as burrowing, incorporate multiple functions. Modified from Hawkes et al. (2017). Reprinted with permission from AAAS. Modified from Blumenschein et al. (2018a) © IEEE 2018, Gan et al. (2020) © IEEE 2020, Agharese et al. (2018) © IEEE 2018, Blumenschein et al. (2018b) © IEEE 2018, Greer et al. (2017) © IEEE 2017, Jeong et al. (2020), Naclerio et al. (2018) © IEEE 2018, Coad et al. (2020a) © IEEE 2020, Slade et al. (2017) © IEEE 2017, and Luong et al. (2019) © IEEE 2019.

While the work discussed in previous sections has investigated methods to understand and expand the capabilities of everting vine robots, here we discuss the previously explored applications for these systems, including the benefits and challenges of using everting vine robots for a given application. Figure 12 shows three main application areas of everting vine robots: deploying and reconfiguring structures, navigating constrained environments, and applying forces on the environment.

### 6.1. Deploying and Reconfiguring Structures

Because everting vine robots create structures as they grow, one area of application has been to create deployable and reconfigurable structures. As discussed in section 3.4.2, sensors and tools can be fixed to the wall of an everting vine robot, allowing controlled deployment and reconfiguration during the growth and steering of the body. In these applications, the shape change of the robot body allows the deployed item to achieve its desired function.

Agharese et al. (2018) designed an everting vine robot to create a deployable wearable haptic device. Haptic devices that modify their surface area are easier to don and doff and can create variable contact depending on the situation. This system begins in a wrist form factor and grows to cover the lower arm, deploying soft pneumatic haptic actuators (Raitor et al., 2017) that provide direction and intensity cues to the wearer. Structure “programability” also allowed for the development of deployable and reconfigurable antennas. In Blumenschein et al. (2018a), copper strips were attached to the robot body wall in an overlapping fashion to form a monopole antenna. As the robot grew and retracted it changed the length of the deployed monopole antenna. Deployment of more complex antenna shapes was shown in Gan et al. (2020), where a handedness-reconfiguring helical antenna was deployed. Other applications that rely on creation of deployable and reconfigurable structures could include deployment of structures in space and the formation of structural metamaterials.

### 6.2. Navigating Constrained Environments

Everting vine robots are well-suited for navigation of constrained environments, especially in situations where non-destructive sensing of the environment and/or delivery of items is needed. The requirements of these applications vary; the goal may be to reach and inspect a particular target with the robot tip, or the robot body itself may be used as a conduit to transport items from its proximal to distal ends, though there is often the additional goal of minimizing the force applied to the environment.

Coad et al. (2020a) reported on the first field deployment of an everting vine robot system in an archaeology application. A portable everting vine robot system was developed that could deliver a camera to collect video inside spaces in an archaeological site that are too small for a human to enter. Due to its ability to navigate tortuous paths, traverse rock blockages, and support its own body through vertical shafts, the everting vine robot was able to collect video in areas previously unobserved by the archaeology team. A similar application area was proposed in Luong et al. (2019), using a water-filled everting vine robot to non-destructively monitor underwater ecosystems. In the field of medicine, preliminary demonstrations have shown the ability of everting vine robots to navigate tortuous paths similar to those encountered inside the human body, with minimal force applied to the environment compared to standard catheters and other medical tools pushed from the base (Slade et al., 2017). Continued work on mounting items at the robot tip without encumbering the robot's navigation ability will enable new capabilities for these types of applications.

In addition to navigating constrained environments through existing paths, everting vine robots can be grown to create a path where no natural pathway already exists. Naclerio et al. (2018) investigated this problem via the development of an everting vine robot capable of burrowing through sand. To adapt the everting vine robot for burrowing, an air line internal to the tail was added to allow for granular fluidization, after which the everting vine robot grew into the sand, using its internal pressure to apply outward forces on the sand to keep its body from being crushed. This combines the navigation and force application abilities of everting vine robots, and it could allow for soil monitoring, non-invasive underground installation, and root-like foundation structures. Another related work (Ozkan-Aydin et al., 2019) showed the benefits of oscillating the everting vine robot tip during navigation of an environment containing both free space and rigid obstacles, similar to how plant roots oscillate their tips when burrowing through soil. This result has also been seen in other growing robot mechanisms (Del Dottore et al., 2017). These designs demonstrate an interesting application of everting vine robots and plant inspired robots in general: as model systems for understanding bio-physical behaviors of plants, similar to how animal inspired robots have been used to better understand animal biophysics (Libby et al., 2012; Li et al., 2013).

### 6.3. Applying Forces

Several potential everting vine robot applications center around applying force on the environment. For example, the natural compliance of an everting vine robot body makes it potentially safe for manipulation around humans. Everting vine robots may be especially useful in environments where a combination of the ability to navigate confined spaces and the ability to apply forces to the environment is needed, such as turning a valve in a disaster scenario (Hawkes et al., 2017).

Moving payloads attached to the robot tip often relies on having sufficient stiffness to resist bending and buckling loads on the everting vine robot body, which depends on the internal pressure and the length of the robot. Because everting vine robots are hollow and filled with fluid, their critical bending and buckling loads tend to be lower than those of traditional robots (section 4.1). Greer et al. (2017) and Stroppa et al. (2020) demonstrated that forces applied using transverse and compressive loading on the everting vine robot body are sufficient to move lightweight objects (200 g) around the robot's 3D workspace, and there is ongoing work on methods to control stiffness (section 3.3.6), which will increase the weight-bearing capacity of everting vine robots to allow the extension to more manipulation tasks.

The use of inextensible materials in many everting vine robots means that, while they tend to be much weaker than traditional robots in compression, they can be strong in tension, and this strength is not dependent on the robot length. Jeong et al. (2020) demonstrated that, with the addition of a tip mount to pull on the environment, everting vine robots can support up to 7 kg of weight and lift up to 2.5 kg in tension, only limited by the strength of the tip mount materials and the tip-mount motors. In a similar application, everting vine robots were used as tensile linear actuators (Abrar et al., 2019).

Finally, everting vine robots can apply forces through the everted body more efficiently than through the everting tip. Pressure has an impressive ability to produce high forces when multiplied by a large area, so, by directly using the internal pressure to apply forces, Hawkes et al. (2017) demonstrated a pneumatic jack capable of growing into a small gap and then lifting over 75 kg, with increasing force capability as the robot grew. Nakamura and Tsukagoshi (2018) applied this lifting capability to design a tool that gently lifts and turns people in bed. Wrapping around objects to grasp them is another common continuum robot behavior that everting vine robots can achieve. Preliminary work on this concept was presented in Blumenschein et al. (2018b), which demonstrates helical grasping.

## 7. Conclusion

Everting vine robots are characterized by their ability to achieve growth through pressure-driven eversion. Within this category are a variety of designs, modeling techniques, control and planning strategies, and application areas. In this review, we summarized and organized much of the recent work on everting vine robots. We highlighted the relative benefits and deficits of everting vine robot design components, from material choice to actuation strategy to sensor and tool delivery method. We also showed the uses for and limitations of existing modeling and control strategies, and we explained application areas by the features of everting vine robots that facilitate them.

With the previous work in everting vine robots in mind, there are a number of open questions in each of the areas discussed. Everting vine robot functionality could be increased through design methods. The majority of everting vine robot materials have been tested at the same scale, so investigation of how these materials function within everting vine robots at much smaller and larger scales is needed. Because the materials are relatively cheap, future exploration of manufacturing methods for mass production of everting vine robots could support the development of vine robot swarms and multi-robot coordination. Within actuation design, future work should include expanding methods for creating complex curves and 3D shapes and investigating actuation strategies to facilitate force control in addition to position control. However, the most pressing area of future research in design is the need to develop methods for attachment of sensors and tools that do not encumber everting vine robots' ability to move through constrained environments and squeeze through gaps smaller than their body cross-section, since these beneficial behaviors are currently difficult to achieve with many of the existing tip mounts described in section 3.4.1.

The biggest gap in modeling for everting vine robots is understanding their dynamic responses and behaviors. A dynamic model could expand the capabilities of everting vine robots, allowing for faster movement and greater force application. Initial work in modeling dynamics for everting vine robots has been completed in simulation (El-Hussieny et al., 2019). Part of developing dynamic models that account for environment interaction involves incorporating more accurate kinematic models for the robots. Continuum models like Kirchoff and Cosserat rod models have been applied successfully to many systems with similar thin flexible form factors in robotics and in graphics (Bergou et al., 2008; Gazzola et al., 2018; Zhang et al., 2019), so adapting these models for everting vine robots is an interesting area of future research. As we continue to use everting vine robots in constrained environments, another area of research in modeling is expanding obstacle interaction models to include compliant obstacles, especially since compliant environments are often the type that require delicate interaction.

Future steps within control and planning should focus on two goals: increasing the ease and functionality of teleoperating everting vine robots, and investigating shared or full autonomy for behaviors that are difficult to achieve under teleoperation. For teleoperation, better robot-level control should be investigated by integrating accurate model-based control methods. In autonomous control, everting vine robot behaviors could be greatly expanded by creating control and planning methods of the body shape and the applied forces. These autonomous behaviors could take inspiration from the tropisms and control strategies seen in natural growth. In all these cases, new sensors that can be incorporated in everting vine robots are needed to sense shape, orientation, or interaction force of the robot, or to measure additional properties of the environment. These sensors may be located at the tip, distributed along the length, or actively re-positioned along the robot. For incorporating these sensors for teleoperation, future studies should look at what sensing modalities and displays give users the best sense of situational awareness. New human interfaces will be needed to allow operators to easily and quickly command more complex, high-level everting vine robot behaviors in teleoperated or shared control.

Lastly, there are many exciting application to explore in the future, many of which can be built based on existing ones. In navigating constrained environments, animal burrows are a well-suited environment to explore using everting vine robots. These burrows are difficult to navigate with existing technology, and everting vine robots could provide a tool to conduct minimally-intrusive population surveying of various species, as well as to gather information on the structures and climates of these underground environments. Everting vine robots have also shown promise in creating or augmenting medical devices (Saxena et al., 2020). Many medical procedures, like colonoscopy and endoscopy, require moving medical devices along existing pathways in the human body, and using everting vine robots could cause reductions in procedure time and reductions in unintended forces applied to the body. Everting vine robots also show great potential in creating tools to aid in search and rescue, due to their ability to move through constrained environments and carry sensors and other payloads. Many other potential applications build into new areas. A growing manipulator, for example, would be able to navigate cluttered human environments while keeping a minimal form factor and then apply forces to pick up or move objects in the environment. Future work for application of everting vine robots will also look at incorporating more actuation and control technologies to yield new behaviors. For example, robot applications that combine navigation of constrained environments and force application through manipulation may require on-demand change of everting vine robot properties to allow low-force application during navigation and high force application during manipulation.

Everting vine robots are a technology still in their infancy. Yet, despite the relatively short time, diverse and interesting applications have been unlocked by their unique abilities. There remain many more questions to understand about their governing physics and how their behaviors can be leveraged and controlled to produce useful technologies, but the work to date has shown that everting vine robots provide a compelling framework through which new soft robotic opportunities can arise.

## Author Contributions

LB defined the scope of this review. LB, MC, and DH wrote the first draft of the manuscript. All authors contributed to manuscript revision and read and approved the submitted version.

## Conflict of Interest

LB, AO, and EH have a pending patent on the combination of growth and steering. LB, MC, and AO have a pending patent on the device for retracting growing robots. The remaining author declares that the research was conducted in the absence of any commercial or financial relationships that could be construed as a potential conflict of interest.
